# Supplementary material for: Structures and functions linked to genome-wide adaptation of human influenza A viruses
Source: Sci Rep. 2019 Apr 18;9:6267. doi: 10.1038/s41598-019-42614-y (PMC6472403; doi:10.1038/s41598-019-42614-y)
Supplement: Supplementary file 1 — Supplementary Information [file 41598_2019_42614_MOESM1_ESM.pdf]

Structures and functions linked to genome-wide adaptation of human influenza A viruses

Thorsten R. Klingen[1], Jens Loers[1], Stephanie Stanelle-Bertram[3], Gülsah Gabriel[3,4] and Alice C. McHardy[1,2†]

[1]Department for Computational Biology of Infection Research, Helmholtz Center for Infection Research, Braunschweig, Germany
[2]German Center for Infection Research (DZIF), Braunschweig, Germany
[3]Heinrich Pette Institute, Leibniz Institute for Experimental Virology, Hamburg, Germany
[4]University of Veterinary Medicine, Hannover, Germany

[†]Correspondence to: Alice.McHardy@helmholtz-hzi.de

Keywords: influenza A virus, positive selection, graph-cut, adaptation

Supplementary Figure 1

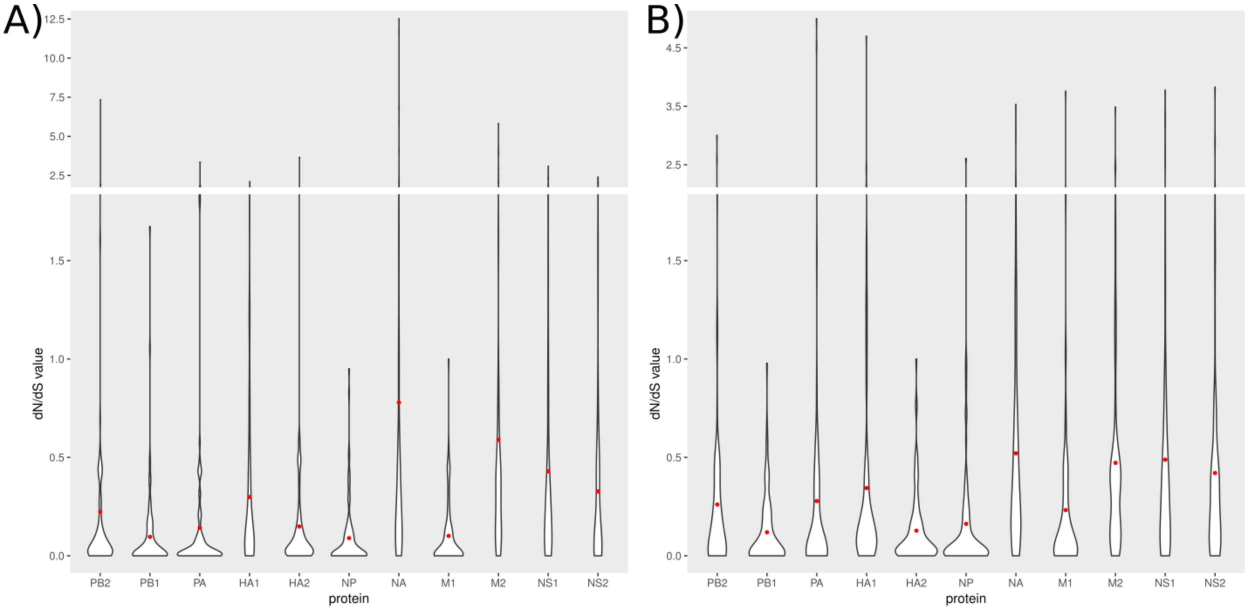

Figure S1: Violin plots of dN/dS distribution.

This figure illustrates the dN/dS distribution in violin plots for H3N2 **(A)** and pH1N1 **(B)** viruses. Red dots indicate the mean dN/dS value.

Supplementary Figure 2

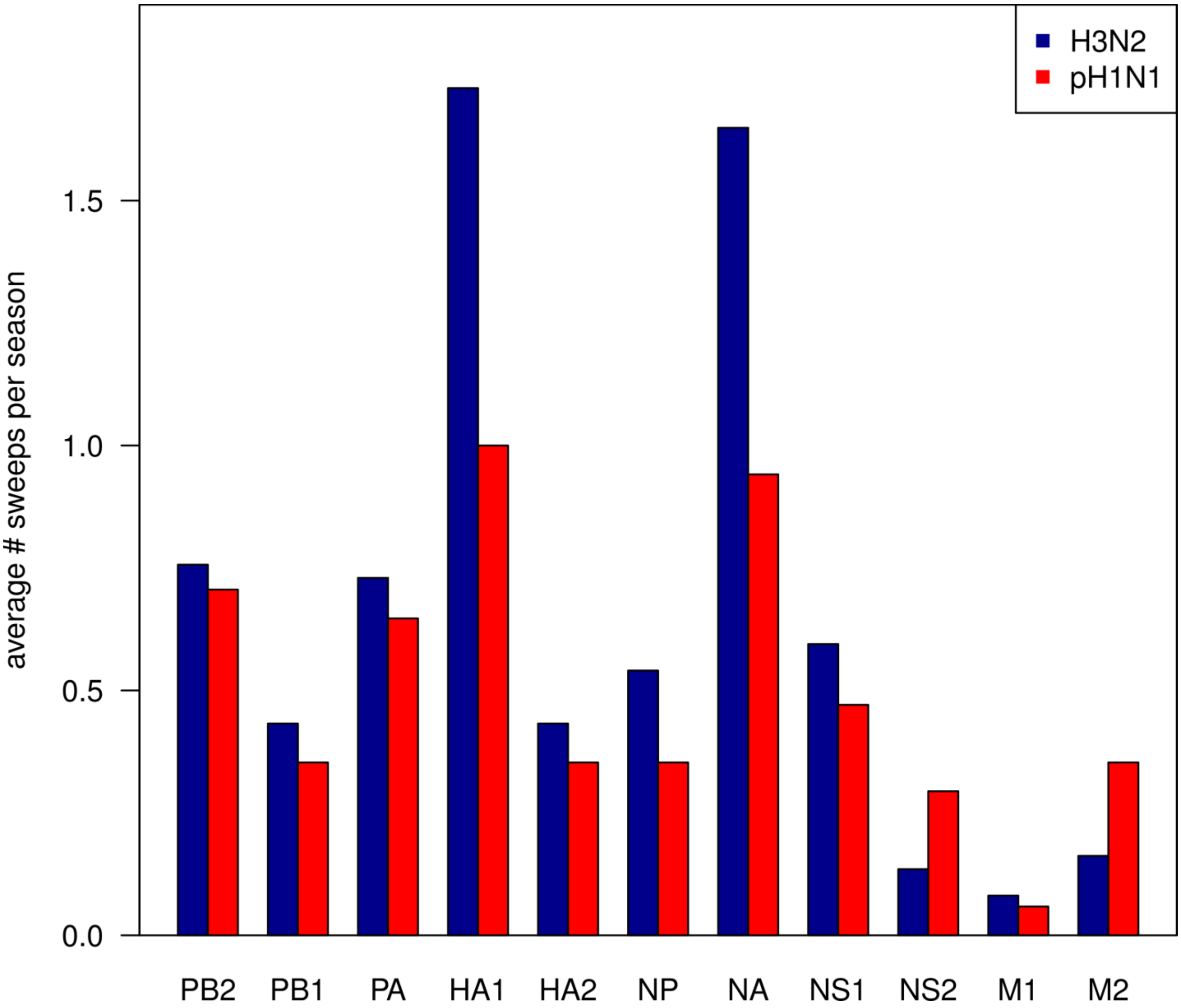

Figure S2: Average number of sweep-related changes.

The barplot show the average number of sweep-related changes per season for all proteins of H3N2 **(blue)** and pH1N1 **(red)** viruses.

Supplementary Table 1

Protein	Target Sequence	Template Structures	Template Region	Template Sequence	Sequence Identity	Region	Coverage
**PB2**	A/California/07/2009(H1N1)	4wsbC	1-482, 491-741	A/little yellow-shouldered bat/Guatemala/060/2010 (H17N10)	67%	1-759	100%
**PB1**		4wsbB	1-194, 199-490, 492-647, 650-733, 736-754		79%	1-757	100%
**PA**		4wsbA	1-185, 204-714		72%	1-716	100%
**HA1**		3m6sA	1-322	A/Darwin/2001/2009 (H1N1)	99%	1-326	99%
		3lzgA	11-325	A/California/04/2009(H1N1)	99%		
**HA2**		3m6B	1-170	A/Darwin/2001/2009 (H1N1)	98%	1-175	100%
		3lzgB	1-175	A/California/04/2009(H1N1)	98%		
**NP**		3ro5A	22-71, 91-198, 212-429, 439-479, 485-497	A/Wilson-Smith/1933 (H1N1)	92%	21-498	96%
		3ro5B	22-83, 88-199, 214-246, 248-393404-428, 441-479, 486-491		92%		
**NA**		4b7QA	83-350, 352-469	A/California/07/2009(H1N1)	100%	83-469	83%
**M1**		3md2A	4-67, 77-85, 89-157	A/California/04/2009 (H1N1)	100%	1-157	62%
		5cqeA	1-71, 73-158	A/Puerto Rico /8/34(H1N1)	95%		
**M2**		2n70A	18, 20-30, 32-49, 51-60	A/Udorn/307/1972(H3N2)	81%	18-62	47%
		2kihA	23-30, 32-49, 51-60		84%		
		2l0jA	22-49, 51-62		83%		
**NS1**		3m5rB	79-203	A/California/07/2009(H1N1)	100%	1-219	100%
		4opaB	2-37, 41, 42-49, 85-203	A/blue-winged teal/MN/993/1980(H6N6)	84%		
		3m8aA	1-72	A/California/07/2009(H1N1)	100%		
**NS2**		1pd3	64-85, 87-103, 105-116	A/Puerto Rico /8/1934(H1N1)	96%	64-114	42%

Table S1: Overview of protein modeling for pH1N1 protein

This table provides detailed information about the homology modeling of all pH1N1 proteins.

Supplementary Table 2

Protein	Target Sequence	Template Structures	Template Region	Template Sequence	Sequence Identity	Region	Coverage
PB2	A/Aichi/2/1968 (H3N2)	4wsbC	1-482, 491-741	A/little yellow-shouldered bat/Guatemala/060/2010 (H17N10)	67%	1-759	100%
PB1		4wsbB	1-194, 199-490, 492-647, 650-733, 736-754		79%	1-757	100%
PA		4wsbA	1-185, 204-714		70%	1-716	100%
HA1		3hmgA	1-225, 227-326	A/Aichi/2/1968 (H3N2)	100%	1-328	100%
HA2		3hmgB	1-175		100%	1-175	79%
NP		4x9aA	21-72, 92-202, 213-396, 403-428, 438-489	A/Wilson-Smith/1933 (H1N1)	91%	21-498	96%
		3zdpB	21-391, 408-498		92%		
NA		3tiaA	82-469	A/RI/5+/1957 (H2N2)	88%	81-469	83%
		2aepA	82-469	A/Memphis/31/98(H3N2)	98%		
M1		1ea3	2-158	A/Puerto Rico/8/1934 (H1N1)	98%	1-160	63%
		5cqeB	1-159		98%		
M2		2ly0A	22-49, 51-62	A/Chiba/5/71 (H3N2)	97%	18-62	47%
		2n70A	18-60	A/Udorn/307/1972(H3N2)	87%		
NS1		1ailA	1-70	A/Udorn/307/1972 (H3N2)	90%	1-230	97%
		4ophA	2-37, 29-40, 42-203	A/blue-winged teal/MN/993/1980 (H6N6)	85%		
		3d6rB	83-202	A/Duck/Alberta/60/1976 (H12N5)	73%		
		3ee9A	84-205	A/Udorn/307/1972(H3N2)	94%		
NS2		1pd3	64-85, 87-103, 105-116	A/Puerto Rico /8/1934(H1N1)	96%	63-116	45%

Table S2: Overview of protein modeling for H3N2 protein

This table provides detailed information about the homology modeling of all H3N2 proteins.

Supplementary Table 3

	H3N2		pH1N1	
	dN/dS	HyPhy SCLAC	dN/dS	HyPhy SCLAC
**PB2**	0.223	0.283	0.260	0.480
**PB1**	0.096	0.204	0.119	0.220
**PA**	0.142	0.212	0.277	0.455
**HA1**	0.298	0.522	0.344	0.506
**HA2**	0.148	0.206	0.127	0.231
**NP**	0.089	0.127	0.162	0.269
**NA**	0.778	0.688	0.520	0.950
**M1**	0.100	0.208	0.232	0.350
**M2**	0.589	1.812	0.472	1.242
**NS1**	0.429	0.589	0.488	0.977
**NS2**	0.327	0.774	0.420	0.611

Table S3: Comparison of mean dN/dS.

As a sanity check, we compared our results with the mean dN/dS value from the Suzuki-Gojobori counting approach implemented in HyPhy SLAC for all proteins.

Supplementary Table 4

protein	p-value
**PB2**	$3 * 10^{-10}$
**PB1**	$2.7 * 10^{-7}$
**PA**	$4.97 * 10^{-9}$
**HA1**	$2.38 * 10^{-2}$
**HA2**	$6.005 * 10^{-1}$
**NP**	$1.46 * 10^{-3}$
**NA**	$1.33 * 10^{-2}$
**M1**	$65 * 10^{-5}$
**M2**	$6.89 * 10^{-1}$
**NS1**	$1.48 * 10^{-1}$
**NS2**	$15.34 * 10^{-4}$

Table S4: Kolmogorov–Smirnov-test.

We applied the Kolmogorov–Smirnov-test (KS-test; H_0: dN/dS distribution of pH1N1 protein is smaller than dN/dS distribution of H3N2 protein) to all proteins individually and showed that the global dN/dS distributions of pH1N1 proteins were significantly larger than those of H3N2 proteins, except for HA2, NS1 and M2.

Supplementary Table 5

protein	p-value
**PB2**	$1.07 * 10^{-10}$
**PB1**	$6.23 * 10^{-7}$
**PA**	$7.17 * 10^{-4}$
**HA1**	$3.59 * 10^{-4}$
**HA2**	0.27
**NP**	$2 * 10^{-2}$
**NA**	0.99
**M1**	$7.28 * 10^{-5}$
**M2**	$2 * 10^{-3}$
**NS1**	$1 * 10^{-3}$
**NS2**	$2 * 10^{-3}$

Table S5: Kolmogorov–Smirnov-test pandemic vs. post-pandemic phase

We applied the Kolmogorov–Smirnov-test (KS-test; H_0: dN/dS distribution of pH1N1 protein during the pandemic is smaller than dN/dS distribution of pH1N1 protein after the pandemic) to all proteins individually and showed that the dN/dS distributions of pH1N1 proteins during the pandemic phase were significantly larger than the dN/dS distributions of pH1N1 proteins in the post-pandemic phase, except for HA2 and NA.

Supplementary Table 6

protein	H3N2	pH1N1
**PB2**	2.503 %	5.270 %
**PB1**	1.453 %	2.113 %
**PA**	2.094 %	4.748 %
**HA1**	16.158 %	15.853 %
**HA2**	1.142 %	0 %
**NP**	0.602 %	3.815 %
**NA**	7.675 %	8.955 %
**M1**	0 %	1.587 %
**M2**	11.340 %	12.371 %
**NS1**	23.043 %	27.397 %
**NS2**	0 %	0 %

Table S6: Frequency of patch sites per protein.

We list the ratio of patch sites to protein sites of the protein model for each protein individually.

Supplementary Table 7

		pH1N1							H3N2					
		Precision	Recall	F-score	Accuracy	Stability	Average dN/dS		Precision	Recall	F-score	Accuracy	Stability	Average dN/dS
**PB2**	1.	0.89865	0.93489	0.91641	0.99938	1	1.79617	1.	0.73694	0.88764	0.8053	0.99897	1	1.1482
	2.	0.90602	0.94085	0.9231	0.99943	1	1.20104	2.	0.80449	0.90809	0.85315	0.99924	1	1.21464
	3.	0.7655	0.90618	0.82992	0.9991	1	1.17321	3.	0.72584	0.9141	0.80916	0.99895	1	1.58804
	4.	0.76449	0.90487	0.82877	0.99909	1	1.91548	4.	1	0.89276	0.94334	0.99878	1	0.98501
	5.	0.76293	0.90597	0.82832	0.99909	1	1.59852	5.	0.80653	0.90255	0.85184	0.99924	1	2.31392
	6.	0.78481	0.91676	0.84567	0.99918	1	1.07023	6.	0.82359	0.91068	0.86495	0.9993	1	1.05223
	7.	0.77196	0.90919	0.83498	0.99912	1	0.72867							
	8.	0.74455	0.89222	0.81173	0.999	0.99136	0.92995							
	9.	0.77124	0.90969	0.83477	0.99912	1	0.73048							
	10.	0.7507	0.9	0.8186	0.99904	1	1.09755							
	11.	0.73232	0.88789	0.80263	0.99895	1	1.05043							
	12.	0.89515	0.92904	0.91178	0.99934	1	1.54116							
	13.	0.75593	0.89884	0.82121	0.99905	1	0.70233							
	14.	0.89947	0.93168	0.91529	0.99937	1	0.91775							
	15.	0.73451	0.89063	0.80507	0.99897	1	4.04162							
	16.	0.74642	0.89878	0.81554	0.99902	1	1.38535							
	17.	0.98371	0.96928	0.97644	0.99977	1	1.10768							
**PB1**	1.	0.819	0.90447	0.85962	0.99928	1	1.46251	1.	0.79183	0.89176	0.83883	0.99918	1	2.71937
	2.	0.795	0.89176	0.8406	0.99919	1	2.07695	2.	0.79582	0.89324	0.84172	0.99919	1	1.17066
	3.	0.607	0.67821	0.64063	0.99816	0.75686	0.56816	3.	0.78486	0.87604	0.82795	0.99912	0.97164	1.347
	4.	0.803	0.89521	0.8466	0.99922	1	2.33944	4.	0.98152	0.59828	0.74341	0.99743	1.99346	1.78638
	5.	0.817	0.90376	0.85819	0.99927	1	1.20138							
	6.	0.90325	0.91508	0.90912	0.99933	1	0.9506							

	7.	0.783	0.88027	0.82879	0.99913	1	1.64516							
**PA**	1.	0.8056	0.89456	0.84775	0.99918	1	1.29466	1.	0.61646	0.93557	0.74321	0.99835	1	0.94922
	2.	0.84558	0.92007	0.88125	0.99935	1	0.73796	2.	0.7854	0.89539	0.83679	0.99911	1	1.59671
	3.	0.81159	0.90272	0.85474	0.99921	1	1.35054	3.	0.62174	0.9416	0.74895	0.99838	1	0.88074
	4.	0.8046	0.89694	0.84826	0.99918	1	1.4778	4.	0.89505	0.65918	0.75922	0.99837	1	1.05252
	5.	0.97066	0.98548	0.97802	0.99983	1	1.29668	5.	0.81995	0.90829	0.86186	0.99925	1	0.60085
	6.	0.89225	0.9011	0.89665	0.99919	1	0.57344	6.	0.80758	0.899	0.85084	0.99919	1	1.50513
	7.	0.97588	0.95062	0.96308	0.99932	1	1.04651	7.	0.40107	0.99945	0.57242	0.99616	1	0.8206
	8.	0.803	0.89571	0.84682	0.99917	1	0.76832							
	9.	0.841	0.91562	0.87673	0.99933	1	1.33694							
	10.	0.92322	0.93484	0.92899	0.99945	1	1.95201							
	11.	0.99614	0.99641	0.99628	0.99996	1	1.7039							
	12.	0.797	0.8945	0.84294	0.99915	1	0.79982							
**HA1**	1.	0.68887	0.88763	0.77572	0.99715	1	1.09228	1.	0.99328	0.95434	0.97342	0.99926	1	0.88453
	2.	0.99257	0.99421	0.99339	0.99985	1	1.59415	2.	0.82742	0.92073	0.87159	0.99771	1	1.21967
	3.	0.7831	0.90859	0.84119	0.99806	1	0.74958	3.	0.68574	0.91079	0.7824	0.99715	1	1.11964
	4.	0.98538	0.86113	0.91908	0.99645	1.02148	1.06979	4.	0.72724	0.83498	0.7774	0.99593	0.88579	0.74254
	5.	0.75755	0.89471	0.82043	0.9978	1	2.15426	5.	0.99642	0.99917	0.99779	0.99995	1	1.23302
	6.	0.96973	0.98811	0.97883	0.99964	1	3.14461	6.	0.99775	0.98032	0.98896	0.99962	1	0.91345
	7.	0.76574	0.90139	0.82805	0.9979	1	4.48946	7.	0.32865	0.5948	0.42337	0.99091	0.62621	0.69681
	8.	0.99136	0.9209	0.95483	0.99875	1	2.31625	8.	1	0.88764	0.94048	0.99601	1	1.33813
	9.	0.99904	0.86652	0.92807	0.99684	1.00924	1.27182	9.	0.65854	0.89158	0.75754	0.99678	0.98301	2.92006
	10.	1	0.94825	0.97344	0.99847	1	1.00421	10.	0.83859	0.92624	0.88024	0.99787	1	2.13886
	11.	0.98451	0.94756	0.96568	0.99923	1	2.19452	11.	0.98462	0.94567	0.96475	0.9986	1	1.18845
	12.	0.75775	0.89766	0.82179	0.99781	1	2.22453	12.	0.65286	0.89832	0.75617	0.99678	1	0.89678
								13.	0.66394	0.90183	0.76482	0.99689	1	0.73665
**HA2**								1.						

**NP**	1.	0.89932	0.93353	0.91611	0.99901	1	0.92948	1.	1	0.93461	0.9662	0.99962	1	1.71105
	2.	0.88013	0.91879	0.89904	0.9988	1	1.88129							
	3.	0.71474	0.88539	0.79097	0.99822	0.99873	0.92798							
	4.	0.96381	0.98411	0.97385	0.99969	0.99728	0.70311							
	5.	0.99357	0.98395	0.98874	0.99982	1	1.02285							
	6.	0.75756	0.90394	0.8243	0.99852	1	1.85452							
	7.	0.76345	0.9148	0.8323	0.99856	1	0.98319							
**NA**	1.	0.97728	0.98998	0.98359	0.99976	1	0.94483	1.	0.9594	0.98852	0.97374	0.99962	1	0.86846
	2.	0.76328	0.90298	0.82727	0.9982	1	1.23092	2.	0.9884	0.99279	0.99059	0.99982	1	2.93439
	3.	0.96974	0.95065	0.9601	0.99924	0.99544	0.81095	3.	0.96802	0.9885	0.97815	0.99969	1	0.73076
	4.	0.70769	0.95267	0.8121	0.99789	1	0.86562	4.	1	0.91162	0.95377	0.99758	1	1.30683
	5.	0.73895	0.89213	0.80835	0.99801	1	1.68588	5.	0.75186	0.89789	0.81841	0.99812	0.99753	0.76207
	6.	0.99955	0.99866	0.99911	0.99998	1	0.96226	6.	0.75105	0.89816	0.81804	0.99812	1	1.89124
	7.	0.75303	0.90028	0.8201	0.99813	1	3.80419	7.	0.99597	0.98429	0.99009	0.99976	1	0.67372
	8.	0.87081	0.91592	0.89279	0.99844	0.99157	1.25152	8.	0.97104	0.94528	0.95799	0.99879	1	2.15367
	9.	0.67306	0.92936	0.78071	0.99751	0.99878	0.75984							
	10.	0.74231	0.90568	0.8159	0.99805	1	2.10581							
	11.	0.55908	0.70719	0.62448	0.99598	0.79151	2.28639							
	12.	0.75012	0.89321	0.81544	0.99808	1	2.8016							
	13.	0.98963	0.97461	0.98207	0.99966	1	1.15015							
	14.	0.98198	0.94913	0.96527	0.99934	1	1.70656							
	15.	0.97456	0.9904	0.98242	0.99975	1	0.8061							
**M1**	1.	0.82067	0.76863	0.7938	0.99534	0.85414	1.25181							
	2.	0.81235	0.76297	0.78689	0.99519	0.85393	2.20936							
**M2**	1.	0.66056	0.97479	0.78749	0.96726	0.97724	1.11922	1.	0.84851	0.98954	0.91362	0.98408	1	1.24919
	2.	0.79189	0.96334	0.86924	0.95693	1	1.55141	2.	0.9532	0.88313	0.91683	0.97572	1	1.8053
	3.	0.33604	0.90235	0.48971	0.92226	0.98922	1.36896							

**NS1**	1.	0.99723	0.99917	0.9982	0.99994	1	1.0147	1.	0.96081	0.99191	0.97611	0.99941	1	1.42656
	2.	0.98196	0.9975	0.98967	0.99965	1	1.39652	2.	0.75012	0.90055	0.81848	0.9968	1	1.15162
	3.	0.86623	0.93089	0.8974	0.9973	1	1.8717	3.	0.99824	0.85413	0.92058	0.99125	1.00844	1.152
	4.	0.99633	0.93063	0.96236	0.99713	1	1.25332	4.	0.9955	0.85718	0.92118	0.99512	1.08145	1.20051
	5.	0.9681	0.99083	0.97933	0.99947	1	1.16333	5.	0.57138	0.8852	0.69448	0.99385	0.99873	1.2421
	6.	0.65033	0.91204	0.75927	0.99512	1	1.60585	6.	0.73052	0.87679	0.797	0.99641	0.96024	2.02527
	7.	0.61437	0.82074	0.70272	0.99415	0.90732	2.26452	7.	0.98704	0.86791	0.92365	0.99151	1	1.25578
	8.	0.98586	0.98668	0.98627	0.99953	1	1.10176	8.	0.61473	0.79867	0.69473	0.99438	0.88049	0.69069
	9.	0.98833	0.84102	0.90874	0.98474	1	1.96486	9.	0.99044	0.98198	0.98619	0.99932	1	2.99836
	10.	0.99812	0.92083	0.95792	0.99754	1	2.5686							
	11.	0.71206	0.90819	0.79825	0.99614	1	2.15917							

Table S7: Overview of all quality measurements per patch.

This is a detailed overview providing the precision, recall, F-score, stability and the average dN/dS of all patches.
